# Supplementary material for: Blocking Periostin Prevented Development of Inflammation in Rhabdomyolysis-Induced Acute Kidney Injury Mice Model
Source: Cells. 2022 Oct 27;11(21):3388. doi: 10.3390/cells11213388 (PMC9658410; doi:10.3390/cells11213388)
Supplement: Supplementary file 1 [file cells-11-03388-s001.zip › cells-1923495-supplementary.pdf]

Supplement Table S1. Linear regression analysis for % Tubular damage score (H-E staining) in the kidney.

% Tubular damage score	Univariate model		*Multivariate model	
	β (95% CI)	p-value	β (95% CI)	p-value
**Periostin**	0.96 (0.69-1.22)	<0.001	-0.21 (-0.51-0.10)	0.171
**WT Day 0**	reference		reference	
**WT rhabdomyolysis Day3**	24.5 (21.5-27.5)	<0.001	28.0 (22.1-33.9)	<0.001
**Pn-null Day0**	-3.63 (-6.60--0.66)	0.019	-4.80 (-8.18--1.43)	0.008
**Pn-null rhabdomyolysis Day3**	3.30 (0.34-6.27)	0.031	2.12 (-1.27-5.51)	0.206

Abbreviations: CI, confidence interval.

* Adjusted for periostin, WT Day 0, WT rhabdomyolysis Day3, Pn-null Day0 and Pn-null rhabdomyolysis Day3.

Supplement Table S2. Primer sets.

Mouse periostin-1 (Pn-1)

Sense 5'-ATAACCAAAGTCGTGGAACC-3'

Antisense 5'-TGTCTCCCTGAAGCAGTCTT-3'

Mouse MCP-1

Sense 5'- ACTGAAGCCAGCTCTCTCTTCCTC-3'

Antisense 5'- TTCCTTCTTGGGGTCAGCACAGAC-3'

Mouse TNF-a

Sense 5'-ACGGCATGGATCTCAAAGAC-3'

Antisense 5'-AGATAGCAAATCGGCTGACG-3'

Mouse IL-6

Sense 5’-CAATGCTCTCCTAACAGATAAG-3

Antisense 5’-CAATGCTCTCCTAACAGATAAG-3

Mouse iNOS

Sense 5’- CAGCTGGGCTGTACAAACCTT-3’

Antisense 5’- CATTGGAAGTGAAGCGTTTCG-3’

Mouse ICAM

Sense 5’-AAACCAGACCCTGGAACTGCAC-3

Antisense 5’-GCCTGGCATTTCAGAGTCTGCT-3

Mouse IL-13

Sense 5’-CAGAGGCCATGCAATATCCTC-3

Antisense 5’-CAGCATGGTATGGAGTGTGGA-3

Mouse IL-10

Sense 5’-GGTTGCCAAGCCTTATCGGA-3

Antisense 5’-GGTTGCCAAGCCTTATCGGA-3
